# Solar power for maternal and neonatal care after the Jajarkot earthquake, Nepal

**DOI:** 10.2471/BLT.25.294930

**Published:** 2026-04-29

**Authors:** Samriddha Rana, Rabin Khadka, Bhoj Raj Bam, Prahlad Dahal, Saugat Pratap KC, Allison Gocotano

**Affiliations:** a World Health Organization Country Office, UN House, Pulchowk, Lalitpur, Nepal.; b Directorate of Health Services, Birendranagar, Nepal.

## Abstract

**Problem:**

A 6.4-magnitude earthquake struck Jajarkot district in Karnali province, Nepal, on 3 November 2023, disrupting electricity supply at essential birthing centres and health posts. The loss of power disabled baby warmers and heating systems, putting mothers and babies at risk.

**Approach:**

The World Health Organization (WHO) Country Office in Nepal conducted rapid facility assessments across eight municipalities and identified three priority birthing centres with critical power deficits. In partnership with the health ministry and local authorities, WHO identified local vendors to install hybrid 5.45 kW photovoltaic systems within 17 days of contract initiation to ensure continuous, off-grid power supply.

**Local setting:**

The three facilities, serving over 7500 people, are located in steep, mountainous terrain. One of the facilities had no grid connection and the two others relied on unreliable seasonal hydropower, which fails during the winter dry season.

**Relevant changes:**

By the end of December 2023, the hybrid photovoltaic systems were operational, providing 24-hour electricity supply. Baby warmers and room heaters were in continuous use, reducing newborn hypothermia risk. Institutional deliveries increased: Pajaru Kha Health Post recorded five deliveries in two weeks following installation compared with none during the preceding winter period (October–December 2023).

**Lessons learnt:**

The installation was facilitated by rapid assessment, early engagement of pre-identified local vendors, and use of existing supply chains and technical capacity. Strong coordination among WHO, the health ministry and local governments enabled timely decision-making. Prior health cluster coordination training, clear governance mechanisms, streamlined approvals and access to emergency funding helped maintain momentum and ensure on-schedule procurement and installation.

## Résumé

**Problème:**

Un séisme d’une magnitude de 6,4 a frappé le district de Jajarkot, dans la province de Karnali, au Népal, le 3 novembre 2023, perturbant l’approvisionnement en électricité des maisons de naissance et des dispensaires essentiels. Cette coupure de courant a mis hors service les couveuses et les systèmes de chauffage, mettant ainsi en danger les mères et les nouveau-nés.

**Approche:**

L’OMS au Népal a mené des évaluations rapides des installations dans huit municipalités et identifié trois maisons de naissance prioritaires présentant des déficits critiques sur le plan énergétique. En partenariat avec le ministère de la Santé et les autorités locales, l’OMS a sélectionné des fournisseurs locaux pour installer des systèmes photovoltaïques hybrides de 5,45 kW dans les 17 jours suivant la signature du contrat, afin d’assurer un approvisionnement continu en électricité hors réseau.

**Environnement local:**

Les trois établissements, qui desservent plus de 7 500 personnes, sont situés sur un terrain montagneux et escarpé. L’un d’eux n’était pas raccordé au réseau, tandis que les deux autres dépendaient d’une énergie hydroélectrique saisonnière aléatoire qui fait défaut pendant la saison sèche hivernale.

**Changements significatifs:**

Fin décembre 2023, les systèmes photovoltaïques hybrides étaient opérationnels et assuraient un approvisionnement électrique permanent. Les couveuses et les radiateurs fonctionnaient en continu, réduisant ainsi le risque d’hypothermie chez les nouveau-nés. Le nombre d’accouchements en milieu hospitalier a augmenté: le dispensaire de Pajaru Kha a enregistré cinq accouchements au cours des deux semaines qui ont suivi l’installation, contre aucun pendant la période hivernale précédente (d’octobre à décembre 2023).

**Leçons tirées:**

L’installation a été facilitée par une évaluation rapide, la mobilisation précoce de fournisseurs locaux préalablement identifiés et le recours aux chaînes d’approvisionnement et aux capacités techniques existantes. Une coordination étroite entre l’OMS, le ministère de la Santé et les autorités locales a permis de prendre des décisions rapidement. Une formation préalable à la coordination du pôle de santé, des mécanismes de gouvernance clairs, des procédures d’approbation simplifiées et l’accès à des fonds d’urgence ont permis de maintenir la dynamique et de garantir un approvisionnement et une installation dans les délais prévus.

## Resumen

**Problema:**

El 3 de noviembre de 2023, un terremoto de magnitud 6,4 sacudió el distrito de Jajarkot, en la provincia de Karnali (Nepal), interrumpiendo el suministro eléctrico en centros de parto esenciales y puestos de salud. La interrupción del suministro eléctrico dejó sin funcionamiento los calentadores neonatales y los sistemas de calefacción, poniendo en riesgo a madres y recién nacidos.

**Enfoque:**

La OMS en Nepal llevó a cabo evaluaciones rápidas de establecimientos en ocho municipios e identificó tres centros de parto prioritarios con déficits críticos de suministro eléctrico. En colaboración con el ministerio de salud y las autoridades locales, la OMS identificó proveedores locales para instalar sistemas fotovoltaicos híbridos de 5,45 kW en un plazo de 17 días desde el inicio del contrato, con el fin de asegurar un suministro eléctrico continuo fuera de la red.

**Marco regional:**

Los tres establecimientos, que atienden a más de 7500 personas, se encuentran en un terreno montañoso escarpado. Uno de los establecimientos no tenía conexión a la red eléctrica y los otros dos dependían de energía hidroeléctrica estacional poco fiable, que falla durante la estación seca invernal.

**Cambios importantes:**

A finales de diciembre de 2023, los sistemas fotovoltaicos híbridos estaban operativos y proporcionaban suministro eléctrico las 24 horas. Los calentadores neonatales y los sistemas de calefacción se utilizaron de forma continua, reduciendo el riesgo de hipotermia en recién nacidos. Los partos institucionales aumentaron: el puesto de salud de Pajaru Kha registró cinco partos en las dos semanas posteriores a la instalación, frente a ninguno durante el periodo invernal anterior (de octubre a diciembre de 2023).

**Lecciones aprendidas:**

La instalación se facilitó gracias a la evaluación rápida, la implicación temprana de proveedores locales previamente identificados y el uso de cadenas de suministro y capacidades técnicas existentes. La estrecha coordinación entre la OMS, el ministerio de salud y los gobiernos locales permitió una toma de decisiones oportuna. La formación previa en coordinación de clústeres de salud, la existencia de mecanismos de gobernanza claros, la agilización de las aprobaciones y el acceso a financiación de emergencia contribuyeron a mantener el impulso y asegurar la adquisición y la instalación conforme al calendario previsto.

## ملخص

**المشكلة:**

ضرب زلزال بقوة 6.4 ريختر منطقة جاجاركوت في مقاطعة كارنالي، في نيبال، وذلك في 3 نوفمبر/تشرين ثاني 2023، مما أدى إلى انقطاع التيار الكهربائي عن مراكز الولادة والمراكز الصحية الأساسية. تسبب انقطاع التيار الكهربائي في تعطيل أجهزة تدفئة الأطفال وأنظمة التدفئة، مما أدي لتعريض الأمهات والأطفال للخطر.

**الأسلوب:**

قامت منظمة الصحة العالمية (WHO) في نيبال بإجراء تقييمات سريعة للمرافق في ثماني بلديات، وحددت ثلاثة مراكز ولادة ذات أولوية تعاني من حالات عجز حادة في الطاقة. بالشراكة مع وزارة الصحة والسلطات المحلية، قامت منظمة الصحة العالمية بتحديد موردين محليين لتركيب أنظمة كهروضوئية هجينة بقدرة 5.45 كيلوواط في غضون 17 يومًا من بدء التعاقد لضمان استمرار إمداد الطاقة خارج الشبكة.

**المواقع المحلية:**

تقع المرافق الثلاثة، والتي تخدم أكثر من 7500 شخص، في منطقة جبلية وعرة. أحد هذه المرافق غير متصل بالشبكة، بينما يعتمد المرفقان الآخران على الطاقة الكهرومائية الموسمية غير الموثوقة، والتي تنقطع خلال موسم الجفاف الشتوي.

**التغيّرات ذات الصلة:**

في نهاية ديسمبر/أيلول 2023، أصبحت أنظمة الطاقة الشمسية الكهروضوئية الهجينة جاهزة للعمل، والتي وفرت إمدادًا كهربائيًا لمدة 24 ساعة يوميًا. كما تم استخدام أجهزة تدفئة الأطفال ومدفئات الغرف بشكل مستمر، مما قلل من خطر انخفاض حرارة الجسم لدى حديثي الولادة. زادت حالات الولادة في المرافق الصحية: سجل مركز Pajaru Kha الصحي خمس حالات ولادة خلال أسبوعين من تأسيس النظام، مقارنةً بعدم تسجيل أية ولادة خلال فصل الشتاء السابق (أكتوبر/تشرين أول إلى ديسمبر/كانون أول 2023).

**الدروس المستفادة:**

تم تسهيل عملية التركيب من خلال التقييم السريع، والتواصل المبكر مع الموردين المحليين الذين تم تحديدهم مسبقًا، والاستفادة من سلاسل التوريد والقدرات التقنية المتواجدة. وساهم التنسيق الفعال بين منظمة الصحة العالمية، ووزارة الصحة، والحكومات المحلية في صنع القرارات في الوقت المناسب. كما أن التدريب المسبق على تنسيق المجموعات الصحية، وآليات الحوكمة الواضحة، وتبسيط إجراءات الموافقة، والحصول على التمويل الطارئ، قد ساعد في الحفاظ على الزخم وضمان إتمام عمليات الشراء والتركيب في المواعيد المحددة.

## 摘要

**问题:**

2023 年 11 月 3 日，尼泊尔卡纳利省贾贾尔科特发生了 6.4 级地震，基础分娩中心和卫生站的供电中断。断电导致婴儿保暖器和供暖系统无法正常运行，使得产妇和婴儿处于危险之中。

**方法:**

世卫组织 (WHO) 尼泊尔办事处对八个市镇的设施进行了快速评估，确定了存在严重的电力短缺的三个优先分娩中心。世卫组织 (WHO) 与卫生部和当地政府合作，选定当地供应商在合同签订后的 17 天内安装 5.45 千瓦混合光伏系统，确保提供持续的离网供电。

**当地状况:**

这三所设施服务超过 7500 人，位于地势陡峭的山区。其中一个设施没有电网连接，另外两个设施依赖不可靠的季节性水电，在冬季旱季无法运行。

**相关变化:**

2023 年 12 月底，混合光伏系统开始运行，提供 24 小时的电力供应。婴儿保暖器和室内暖气也在持续使用中，降低了新生儿体温过低的风险。机构分娩量增加：帕贾鲁卫生站记录显示，与冬季旱季期间（2023 年 10 月至 12 月）无一例分娩相比，在安装后两周内有五例分娩。

**经验教训:**

此次安装得益于快速评估、尽早接洽预先确定的当地供应商，以及使用现有的供应链和技术能力。世卫组织 (WHO)、卫生部及地方政府间强有力的协调使得及时决策成为可能。之前的卫生群组协调培训、清晰的管理机制、简化的审批流程以及应急资金的获取，帮助维持工作势头，确保能够按计划采购与安装。

## Резюме

**Проблема:**

3 ноября 2023 года в районе Джаджаркот (провинция Карнали, Непал) произошло землетрясение магнитудой 6,4 балла, в результате чего было нарушено электроснабжение жизненно важных родильных центров и пунктов оказания медицинской помощи. Отключение электроэнергии вывело из строя обогреватели для новорожденных и системы отопления, поставив под угрозу здоровье матерей и младенцев.

**Подход:**

Бюро ВОЗ в Непале оперативно провело оценку учреждений в восьми муниципалитетах и определило три приоритетных родильных центра с критическим дефицитом электроэнергии. В партнерстве с Министерством здравоохранения и местными властями ВОЗ определила местных подрядчиков для установки гибридных фотоэлектрических систем мощностью 5,45 кВт в течение 17 дней с момента заключения контракта, чтобы обеспечить непрерывное автономное электроснабжение.

**Местные условия:**

Эти три учреждения, обслуживающие более 7500 человек, расположены в труднодоступной высокогорной местности. Одно из учреждений не было подключено к электросети, а два других зависели от ненадежной сезонной гидроэнергетики, которая перестает работать в засушливый зимний сезон.

**Осуществленные перемены:**

К концу декабря 2023 года гибридные фотоэлектрические системы были введены в эксплуатацию и обеспечивали круглосуточное электроснабжение. Обогреватели для новорожденных и системы отопления помещений использовались в непрерывном режиме, что снизило риск гипотермии у новорожденных. Количество родов в учреждениях возросло: в пункте оказания медицинской помощи Паджару Кха за две недели после установки оборудования было принято пять родов, тогда как за предыдущий зимний период (с октября по декабрь 2023 года) не было зафиксировано ни одних.

**Выводы:**

Установке оборудования способствовали: быстрая оценка, раннее привлечение предварительно отобранных местных подрядчиков, использование существующих цепочек поставок и технического потенциала. Тесная координация между ВОЗ, Министерством здравоохранения и местными органами власти обеспечила своевременное принятие решений. Предварительное обучение координации кластеров здравоохранения, четкие механизмы управления, упрощенные процедуры согласования и доступ к экстренному финансированию помогли сохранить темп работы и обеспечить своевременные закупки и установку.

## Introduction

Nepal is highly vulnerable to natural disasters due to its mountainous terrain and seismically active location.^1^ On 3 November 2023, during the Himalayan winter, a 6.4 magnitude earthquake struck Jajarkot district in Karnali province, killing 154 people. The effect was immediate and catastrophic,^2^ with dozens of health facilities either destroyed or rendered nonfunctional, and thousands of people displaced to temporary shelters.^3^ Within days, the Nepal Ministry of Health and Population requested help from the World Health Organization (WHO) to continue essential health services in the affected districts.

Among the most pressing challenges was the disruption of electricity supply to birthing centres and health posts, rendering neonatal warmers used for preventing hypothermia-related neonatal death nonfunctional.^4^ Although these health facilities occasionally experience poor electricity supply, damage to electric lines further worsened the situation. Furthermore, because their houses were destroyed, mothers and their babies who would normally have been discharged needed to stay at the birthing centres, increasing service demand.

In response to the emergency, the WHO Country Office in Nepal prioritized rapid assessment and response in municipalities with considerably affected birthing services. Systemic power shortages in these municipalities highlighted the need for an alternative sustainable power solution to restore vital maternal and child health services. Here, we describe the lessons learnt from implementing hybrid solar–battery systems to ensure uninterrupted power for neonatal care in three selected health facilities.

## Local setting

The health-care system in Nepal is based on a decentralized framework, with primary health care provided at rural health posts and birthing centres, particularly in remote and mountainous provinces.^5^ Jajarkot district, which is located in Nepal’s mid-western hills, is among the country’s most remote and socioeconomically disadvantaged districts.^6^ The hilly terrain, dispersed settlements and limited road connectivity pose obstacles to health service access. The weak health infrastructure relies on small-scale, run-of-river hydropower schemes or grid connections that regularly fail during the dry season.

All three facilities selected for the intervention had unreliable electricity supply even before the earthquake. The Pajaru Kha Health Post in Chhedegad municipality had never been connected to the national grid, and Limsa Basic Hospital in Barekot and Gharkhakot primary health care in Junichande relied on seasonal micro-hydropower, which was already unreliable and became almost nonfunctional during winter due to decreased water flow. During power outages, essential equipment, such as baby warmers and room heaters, failed to operate, putting mothers and newborns at risk.^7^ Health workers resorted to using hot water bottles and warm clothing to maintain newborn body temperature during these outages. 

## Approach

The intervention drew on WHO’s experience of procuring and installing a 150 kW on-grid photovoltaic system at Province Hospital Surkhet in Karnali province,^8^ where local solar vendors had already been identified and engaged, enabling rapid needs assessment, procurement and installation. 

During the second week of November 2023, the WHO Nepal Health Emergency Programme conducted a quick survey (approximately 5–7 days) of Jajarkot’s eight municipalities to determine service disruption and infrastructure damage caused by the earthquake. In consultation with the local governments, the assessment team identified three high-priority birthing centres where electricity shortages were a critical bottleneck for maternal and neonatal services. The selected facilities included the Limsa Basic Hospital in Barekot, the Pajaru Kha Health Post in Chhedegad and the Gharkhakot primary health-care centre in Junichande. Based on the assessment and guided by WHO’s *Health emergency and disaster risk management framework*,^9^ which promotes sustainable, context-specific and resilient solutions in emergencies, the WHO Country Office prioritized a context-appropriate solar energy solution. The WHO Country Office deemed this solution to be a feasible, sustainable and rapidly deployable option to ensure continuity of essential health-care functions for these three facilities. Through coordination, local, provincial and federal government authorities validated the selected solution.

WHO also facilitated coordination with the Provincial Health Emergency Operations Centre^10^ and health cluster partners,^11^ by supporting a needs communication process. Power shortages identified at health posts were reported to the municipality level and escalated through provincial and federal levels before validated requests were communicated to WHO. This approach ensured validation of priorities, avoided duplication and aligned the intervention with broader earthquake recovery efforts for essential maternal and neonatal services.

The hybrid photovoltaic solar system was jointly selected by the Ministry of Social Development in Karnali province, health ministry and the WHO Country Office, because of its suitability for remote off-grid sites.^12^ Each facility received a 5.45 kW system containing battery storage with a capacity of more than 24 hours. Procurement was conducted by the WHO Country Office through a competitive request-for-quotation process involving three local vendors; submitted quotations were evaluated and the most technically compliant and responsive vendor was selected to supply, transport and install the systems. The selected vendor completed all installations within 17 days of contract initiation, in accordance with the timelines specified in the procurement requirements. WHO personnel supervised the installation and verified compliance with safety and performance standards. To simplify facility management and ensure continued system functionality, the WHO Country Office included a three-year, no-cost maintenance arrangement with the selected vendors as a part of the procurement package.

To ensure local ownership, WHO collaborated with municipal authorities, health facility management and the health ministry to conduct staff orientation on system operation, basic energy management, routine maintenance practices and troubleshooting, with emphasis on prioritizing power for essential maternal and neonatal services. Community sensitization activities included informing patients at consultations about the improved availability of reliable electricity, its role in safe deliveries and newborn care and encouraged timely care-seeking and shared responsibility for safeguarding the solar power systems. 

Following installation, WHO technical staff conducted monitoring visits to each facility to assess solar system functionality, verify performance through functional testing, and provide supportive supervision. These visits included on-site inspection of the photovoltaic and battery systems, discussions with facility staff members on system use, and reviews of solar power integration into routine service delivery.

## Relevant changes

Within days of the solar system installations, previously nonfunctional baby warmers were in full use and provided thermal protection for newborns. Measured voltage output and the uninterrupted operation of essential equipment, including baby warmers, confirmed that power supply was sufficient for the intended purpose. Through regular monitoring, the WHO team confirmed continued system functionality and appropriate use of electricity for priority health services, in line with the staff orientation and without overloading the systems. As of December 2025, the health facilities had not reported any power-related interruptions.

Health workers from all three health facilities reported improved working conditions, more reliable lighting for night deliveries and improved working motivation due to the consistent supply of electricity. They also reported, along with patients, that the information on electricity availability was reinforced during routine patient interactions, helping to raise community awareness of the facility’s improved service readiness and its ability to provide uninterrupted maternal and emergency care.

The intervention reached a catchment population of over 7576 people. Pajaru Kha Health Post recorded five deliveries within two weeks post-installation compared with none during the preceding winter period (October–December 2023). By December 2025, the primary health-care centre in Gharkhakot had reported 90 births, Pajaru health post had recorded 89 births and the Basic Hospital at Limsa had reported 278 births since the installation, illustrating continuity of service provision.

## Lessons learnt

These experiences show how local and renewable energy solutions can ensure continuity of essential services during emergencies and, in parallel, increase health system resilience in underserved, disaster-prone communities. The main lesson learnt from the intervention is that a stable power supply directly enables the continuous functioning of essential clinical services (Box 1). The off-grid solar and battery system was a rapid, sustainable intervention that operated reliably through the winter and addressed seasonal hydropower outages in a resource-poor, disaster-affected environment.

Box 1 **Summary of main lessons learnt**

- Installation of off-grid hybrid photovoltaic and battery systems provides a rapid and sustainable solution for restoring essential health services in disaster-affected, resource-poor settings.
- Early site assessment and use of local vendors accelerate implementation in remote areas with limited access.
- Pre-existing coordination mechanisms and prior health cluster training enable faster decision-making and more effective collaboration across government levels and partners.

Key facilitators for a swift implementation were rapid evaluation and involving pre-identified local solar vendors and service providers to support expedited procurement, transportation and installation using existing local supply chains and technical capacity. Close coordination between the WHO Country Office, the health ministry and rural municipality governments enabled decision-making and resource mobilization in a timely manner. Previous health cluster coordination training for officials involved in health emergency operations, coordination and response planning, proved valuable, as approvals, procurement and installation were completed according to schedule. Implementation records showed that strong institutional leadership and established governance mechanisms, including streamlined approvals, access to emergency funding and clear technical guidance, helped maintain momentum and ensured that procurement and installation followed the planned timeline.

We encountered some implementation challenges. First, the limited baseline data on equipment load and operating hours, necessitated WHO engineers making on-site assessments to determine the appropriate capacity of solar panels and battery storage needed to meet facility electricity demand for priority maternal and neonatal services. These assessments required additional technical input but did not significantly delay the project timeline. Second, the remote, mountainous terrain and limited road connectivity delayed transportation of solar equipment, extending installation by approximately 13 days compared with more accessible conditions, where installation could be completed within 3–4 days. These delays were mitigated through close coordination with local vendors and adjusted logistical arrangements. 

The total costs for procuring and installing the photovoltaic systems at the three health facilities were 36 568 United States dollars. The South-East Asia Regional Health Emergency Fund provided funding, which enabled expedited procurement and installation under emergency response mechanisms. The relatively low investment in renewable energy to improve health-care services can inform WHO’s broader agenda for sustainable health infrastructure.

This project illustrates the potential of renewable energy to strengthen health system resilience, particularly in off-grid and vulnerable settings.^13^ In post-earthquake Jajarkot, the installation of solar power systems directly decreased the risk of power loss in rural health posts. The hybrid photovoltaic systems not only met the immediate clinical needs of mothers and newborns during the winter, but also developed sustainable capacity within the health system. This experience emphasizes renewable energy integration as a viable, high-impact strategy for continuity of care in crisis settings. Furthermore, the implementation model is scalable and generalizable, relying on adaptable components that can be replicated in other off-grid or disaster-prone contexts with similar infrastructure constraints and seasonal power deficits.
